# Refining Housing, Husbandry and Care for Animals Used in Studies Involving Biotelemetry

**DOI:** 10.3390/ani4020361

**Published:** 2014-06-19

**Authors:** Penny Hawkins

**Affiliations:** Research Animals Department, Royal Society for the Prevention of Cruelty to Animals (RSPCA), Wilberforce Way, Southwater, West Sussex, RH13 9RS, UK; E-Mail: penny.hawkins@rspca.org.uk; Tel.: +44-300-123-0231

**Keywords:** Three Rs, refinement, reduction, biotelemetry, environmental enrichment, animal husbandry, surgery, ethical review, animal welfare, welfare assessment

## Abstract

**Simple Summary:**

Biotelemetry, the remote detection and measurement of an animal function or activity, is widely used in animal research. Biotelemetry devices transmit physiological or behavioural data and may be surgically implanted into animals, or externally attached. This can help to reduce animal numbers and improve welfare, e.g., if animals can be group housed and move freely instead of being tethered to a recording device. However, biotelemetry can also cause pain and distress to animals due to surgery, attachment, single housing and long term laboratory housing. This article explains how welfare and science can be improved by avoiding or minimising these harms.

**Abstract:**

Biotelemetry can contribute towards reducing animal numbers and suffering in disciplines including physiology, pharmacology and behavioural research. However, the technique can also cause harm to animals, making biotelemetry a ‘refinement that needs refining’. Current welfare issues relating to the housing and husbandry of animals used in biotelemetry studies are single *vs*. group housing, provision of environmental enrichment, long term laboratory housing and use of telemetered data to help assess welfare. Animals may be singly housed because more than one device transmits on the same wavelength; due to concerns regarding damage to surgical sites; because they are wearing exteriorised jackets; or if monitoring systems can only record from individually housed animals. Much of this can be overcome by thoughtful experimental design and surgery refinements. Similarly, if biotelemetry studies preclude certain enrichment items, husbandry refinement protocols can be adapted to permit some environmental stimulation. Nevertheless, long-term laboratory housing raises welfare concerns and maximum durations should be defined. Telemetered data can be used to help assess welfare, helping to determine endpoints and refine future studies. The above measures will help to improve data quality as well as welfare, because experimental confounds due to physiological and psychological stress will be minimised.

## 1. Introduction

The ethical decision-making process for research projects or procedures involving animals rests on a harm-benefit assessment, in which the likely harms (pain, suffering or distress experienced by animals) are ‘weighed’ against the potential benefits of the project. This assessment may be conducted by a number of different individuals or bodies, including the regulator, an ethics committee, the researcher or a combination of these. A good harm-benefit assessment involves setting out the potential harms to animals throughout their lives, including not only scientific procedures and their after effects, but also other aspects that may cause suffering such as early ‘weaning’, transport, husbandry restrictions (e.g., singly housing social animals) and some killing techniques. This approach also identifies opportunities to implement refinement, one of the Three Rs (Replacement of animal experiments with humane alternatives, Reduction of animal numbers to the minimum needed for statistical significance, and Refinement of animal husbandry and procedures to reduce suffering and improve welfare). Refinement can significantly reduce harms to animals, which will clearly impact on the harm-benefit assessment and have implications as to the justification for the project. However, refining one aspect of a procedure may have repercussions that affect welfare in other ways, so it is essential to conduct a thoughtful review of the animal’s broader life experience when making decisions about appropriate refinements and how these will be implemented.

One example of a refinement that can give rise to wider welfare issues is biotelemetry, which can be defined as the remote detection and measurement of a human or animal function, activity or condition [1]. The first physiological signal telemetered from a conscious animal was recorded in 1963, when aortic blood flow was successfully transmitted from an exercising boxer dog at the San Diego Zoo hospital [2]. Since then, the use of biotelemetry has expanded hugely within a wide range of biological disciplines including physiology, pharmacology and behavioural studies in both the laboratory and field [3]. Its use has become so well-established that some regulatory bodies require data that can only be obtained using biotelemetry [4].

The use of biotelemetry to transmit physiological or behavioural data is often cited as a way of implementing two of the Three Rs; reduction and refinement. Biotelemetry can indeed reduce animal numbers, by enabling the collection of more, better quality data from each animal, e.g., because data can be gathered over longer periods, or multiple parameters can be recorded from the same individual [3,5]. Removing the need for tethers and restraint during recording, so that animals can behave more normally, is a refinement in that it can significantly reduce stress during data collection [5].

However, despite these benefits, the application of biotelemetry can also be a source of harms to animals. Surgical procedures to implant internal devices can cause pain and distress, as can the fitting of external devices, and the size, shape and weight of the device can be a welfare issue in smaller species [5]. Recording multiple parameters may add to the burden on the animal (e.g., if multiple sensors are implanted), and holding animals in the laboratory in the long term is also a cause for concern and debate as to acceptable maximum durations. Animals may be singly housed, either during recordings or permanently, which is a significant welfare issue for social species or strains.

Taking all of this into account, the use of biotelemetry within each project should **in itself** be subject to a harm-benefit assessment, in common with all other invasive procedures. It is essential to recognise this and regard biotelemetry as a ‘refinement that may need refining’ rather than a panacea. With this in mind, the RSPCA set up an expert Joint Working Group on Refinement (JWGR) in 2003 to produce good practice guidance reports on procedures involving biotelemetry [5] and housing and care for animals used in such projects [6]. This was within the context of the Society’s work to promote effective ethical review of animal use and implementation of the Three Rs.

Ten years on, the use of telemetry has continued to increase, as have developments in device technology. This paper provides an update on some of the topics addressed in the JWGR on housing, husbandry and care for animals involved in biotelemetry studies. It does not address device attachment or implantation, but assumes that these will be fully refined in accordance with legal requirements to minimise suffering by employing effective pain management [7] and current good practice approaches to surgery such as the Laboratory Animal Science Association (LASA) asepsis guidelines [8].

Also over the last decade, the level of debate has increased within the scientific community about the scientific validity and translatability of many animal ‘models’. Concerns that have come to the fore include the validity of ‘extrapolating’ between species, standards of experimental design and reporting, and confounding effects due to physiological and psychological responses to housing, husbandry and care that does not cater for animals’ species-specific needs [9,10,11,12,13,14]. All of these issues have profound ethical implications with respect to the actual ‘benefits’ of animal research, how funding resources are spent, and whether the needs of patient groups are properly addressed. Reducing stress to animals by employing the approach to housing and care set out in this document will thus not only help to improve welfare, but will also enhance scientific validity.

## 2. Housing, Husbandry and Care Issues Associated With Biotelemetry

A refined biotelemetry system should: allow animals to be housed in stable, compatible groups; not preclude environmental enrichment; be minimally invasive; and would not be interfered with by other animals in the group. In practice, however, internally and externally mounted devices currently both facilitate and present obstacles to these standards [5,6]. This is expanded upon in Section 2.1 and Section 2.2 below, which explain how to provide group housing and enrichment for animals on biotelemetry studies. Some instrumented animals are reused and housed long term, which is addressed in Section 2.3, while Section 2.4 provides examples of how available telemetered data can be used for an additional purpose: to help reduce suffering and improve welfare.

### 2.1. Group Housing

It is generally accepted that social species, including many commonly used laboratory mammals, should be housed in stable, compatible groups [7,15]. This varies with species, strain and sex, for example there is debate over the benefits of group housing hamsters and some strains of male mice. Singly housing social species or strains is likely to have significant negative consequences for animal welfare and will also affect data quality, if social isolation leads to physiological responses that affect results [16,17,18,19,20,21]. Despite this, a proportion of animals used in biotelemetry studies are still singly housed. The reasons that are given for this should always be challenged, as many of the perceived obstacles to group housing can be overcome, as explained below.

**Reason 1: Devices all transmit on the same wavelength.** Although considerable developments have been made in this area in recent years, many commercially available telemetry devices still transmit at the same frequency. It is obviously impossible to obtain meaningful data from several devices, on different animals, that are all transmitting at the same wavelength within the same pen or cage. If the use of single wavelength devices is unavoidable, solutions include:

(1) The ‘buddy’ system, in which an instrumented animal is group housed with one or more uninstrumented individuals [20,21]. The fate of the buddy (or buddies) can be planned for in advance. Potential options are: (i) rehoming; (ii) use in another study, provided that the animals are suitable (e.g., of an appropriate age); or (iii) humane killing at the end of the study. Both options (i) and (ii) may involve changing established groups, in which case advice from animal technologists or the attending veterinarian can help to reduce stress to the animals. Option (iii) is an ethical issue, although it need not be an animal welfare issue if the killing technique is properly refined. The animals’ tissues or organs should be used in other studies or for training purposes where appropriate, or facilities may sell surplus, euthanased animals for use as food for rehabilitated wildlife, raptors or ‘exotic’ companion animals. Some establishments have set up programmes in which surplus animals can be certified fit and rehomed to responsible carers [22,23] and there is a legal framework to support rehoming in many countries, provided that it is in the animal’s best interests [7].
(2) Devices that can be switched on and off with a magnetically actuated system *in situ*, *i.e.*, following implantation, and used one at a time in pair- or group-housed animals. This avoids the issue of deciding fates for ‘ex-buddies’, provided that recording data sequentially is compatible with the scientific protocol, or can be compensated for by the experimental design. Switching devices on and off can also extend battery life, thus facilitating reduction in animal numbers because more data can be gathered from each animal (but see Section 2.3 below).
(3) Instrumenting all animals, and then separating them for data recording sessions only. This allows data to be recorded at the same time from different individuals, but periods of social isolation (and being moved to a different enclosure, if applicable) will be highly stressful for some animals, with implications for welfare and data quality [20,21]. The suitability of this approach will depend upon factors such as the species, duration of isolation and whether animals can habituate to the protocol.
(4) Use of data logging devices instead of transmitters, where data are recorded onto a microchip and downloaded once the device has been retrieved from the animal. Data can be retrieved following each session if external loggers are used, but there will clearly be a longer wait in the case of implanted loggers. Also, device failure may not be detected until the download is attempted, meaning that studies would have to be repeated.

At the time of writing, biotelemetry device manufacturers are working on technical innovations that will further facilitate pair and group housing, so it is good practice to keep up to date with developments in multi-wavelength devices and other innovations that can reduce the impact of telemetry on animals, e.g., smaller or lighter devices. A wider range of dual frequency and multi-channel devices has become available; initially for large animals such as dogs, but some are now marketed as small enough for use in rats. However, there are currently no commercially available, implantable, multi-wavelength devices that are suitable for mice.

**Reason 2: Animals have undergone implantation surgery and are singly housed in case they damage one another’s wounds.** Surgical wound failure is painful and distressing, affecting both health and welfare and requiring either repair surgery or the euthanasia of the animal. However, the negative welfare impact of changing from social to permanent single housing should not be underestimated for social species. To look at this another way; wound damage or failure may occur, but the psychological distress due to changing to single housing will occur. In light of this, the ideal is to re-establish animals in their social groups as soon as possible following any kind of surgery, and this should also be the default position for biotelemetry [6].

Different facilities employ a variety of protocols with respect to regrouping after implantation surgery, ranging from immediate regrouping to waiting for a period of several days. In general, animals can be successfully regrouped after 24 hours [5] if steps are taken to ensure that:

- The pair or group was well-established and stable before surgery. If groups are stable before surgery, there is a greater chance of achieving harmonious regrouping afterwards. Drawing pairs or groups from littermates is an obvious option, which should be possible whether animals are bred either in-house or externally. Good liaison with the breeder will be necessary if animals are sourced from outside the establishment (liaison with any external breeding facilities to ensure consistency in housing and care conditions is also good practice, although if there are differences then the aim should be to ‘level up’ and implement the better husbandry protocols at both).
- If animals have undergone transport to the facility, they have had an adequate settling-in period to enable both recovery from any transport stress and acclimatisation to environmental changes and new caretakers. Absolute minimum settling-in periods before surgery have been suggested of a week for rodents, two weeks for dogs, and a month for non-human primates [6]. Note that animals should be allowed a minimum post-implantation recovery period of two weeks before conducting further procedures [20,21].
- Surgical procedures represent best practice with respect to asepsis, surgical approach, the competence of the surgeon and effective welfare assessment and pain management [5,8,24]. All of these will facilitate a more rapid return to normal behaviour, including social behaviour.
- Animals have recovered from anaesthesia so that they are capable of interacting appropriately with others.
- Wound closure has been fully refined, for example by the use of intradermal sutures [24]. This, together with effective pain management and careful asepsis, will stop animals paying excessive attention to their own wounds, reducing the risk of drawing the attention of others *via* visual or olfactory cues.
- Housing and care are also refined, including the provision of sufficient space and enrichment, which will shift animals’ attention from their own and others’ wounds and allow them to retreat from one another [6].
- There is adequate supervision from carers in the initial regrouping period, and for as long as is necessary to ensure that there is no persistent discomfort or pain. Behavioural assessments should also check that animals are interacting in an acceptable way, e.g., hierarchies are appropriate and there is no bullying [5,25].

There may be concerns about wound breakdown and bullying if group housing has not been usual practice, or if there is a proposal to regroup animals earlier following surgery. In these cases, a carefully-supervised pilot study, with advice from the attending veterinarian, could be undertaken. This should include and address all of the points in the above list.

One approach to reducing any negative effects of single housing that might arise is ‘living apart together’, in which animals have no (or minimal) physical contact with one another but can see, hear and/or smell other animals. Visual and olfactory contact is recommended for other species such as non-human primates, rabbits and dogs when separately housed [6,26,27,28]. However, the benefits of this limited contact can vary between species and strains. For example, female C57BL/6JOlaHsd mice benefit most from social housing after surgery, but if they are separated, limited contact through a grid partition is more stressful than housing them individually [29]. The literature on the effects of social *vs*. individual housing, including behavioural studies to evaluate whether particular species and strains benefit from limited contact, should be regularly reviewed to ensure that protocols reflect current knowledge about animal behaviour and welfare.

*If single housing really is unavoidable for justifiable scientific reasons, animals should not undergo implantation surgery unless they are able to tolerate being housed on their own. A trial period should be set up to assess how well animals can adapt before moving on with the study.*

**Reason 3: Animals are wearing jackets or have exteriorised devices and are singly housed in case they interfere with these.** Similar issues arise when group housing animals with devices that are partly or entirely external as when following surgery. Some species can be group housed while fitted with external devices without problems, but with other species there may be concerns that animals could damage one another’s jackets or devices, or that stress or injuries could occur due to excessive attention to these. The basic approaches to overcoming this are the same as those for group housing post-surgery, *i.e.*, maintaining stable groups, refining housing and care, and ensuring appropriate supervision.

Other measures that can be undertaken to facilitate group housing for animals with jackets, harnesses or exteriorised devices include ensuring that animals are fully habituated to the device and/or attachment, are trained to accept it and are also accustomed to other animals wearing it. Animals may habituate to relatively large exteriorised devices if they are given the opportunity. For example, mice with headpieces measuring 15 by 20 mm have been successfully housed in groups of 12, with no injuries to the animals or significant damage to any of the devices. This was facilitated by forming and maintaining stable groups and by adding non-working devices to the cage for the mice to explore and manipulate. Although some of the mice tried to bite others’ devices for a period of 15 to 20 minutes when first re-grouped after the headpieces were fitted, this was not sustained and the animals quickly lost interest [30].

For studies where jackets are required, trials can be conducted using old jackets and dummy devices to help with habituation. If the only problem lies with animals undoing fastenings, these can simply be made more robust. Some animals (e.g., some groups of dogs, particularly very young ones) may persist in damaging one another’s jackets and/or devices, so that it really is impossible to group house them. In such cases, any welfare impact can be reduced by minimising the duration of single housing and implementing ‘living apart together’ for those species that will benefit from this (see above). It should also be possible to allow socialisation time for singly housed animals in a shared area, with adequate supervision and activities to distract from one another’s jackets. Given the wide availability and increasing use of jacketed electrocardiogram (ECG) telemetry systems in large animals, achieving group housing for these animals is a very important goal for animal welfare.

**Reason 4: Cage-type monitoring systems that can only record from singly housed animals are being used.** Automated, cage-based monitoring systems are sometimes used for behavioural monitoring, for example in phenotyping screens or in neuroscience studies. These may operate using video, by recording food and water consumption, by recording and analysing an animal’s movements within a cage placed on a sensor platform, or by a combination of these. Some of these monitoring systems cannot record from or track multiple animals, which necessitates single housing. It is somewhat paradoxical for a behavioural monitoring and characterisation system to require animal housing that is inherently stressful and liable to lead to confounding results, so the use of such systems should be critically questioned on both animal welfare and scientific grounds.

There are some cage-based monitoring systems already available that use transponder technology to recognise individuals, or that include software capable of distinguishing between animals. Where possible, these should be used in preference to those that only permit single housing. As with biotelemetry device technology, it is important to monitor ongoing developments and regularly check whether there is an alternative product or option that permits group housing for social animals.

### 2.2. Providing Environmental Enrichment

Animals with exteriorised devices are sometimes subject to restrictions on environmental enrichment for fear that they will become entangled or entrapped in some enrichment items (e.g., some nesting materials or refuges). Concerns that animals might hurt or injure themselves on some items can also result in reduced provision of enrichment for animals on biotelemetry studies.

**Figure 1:**
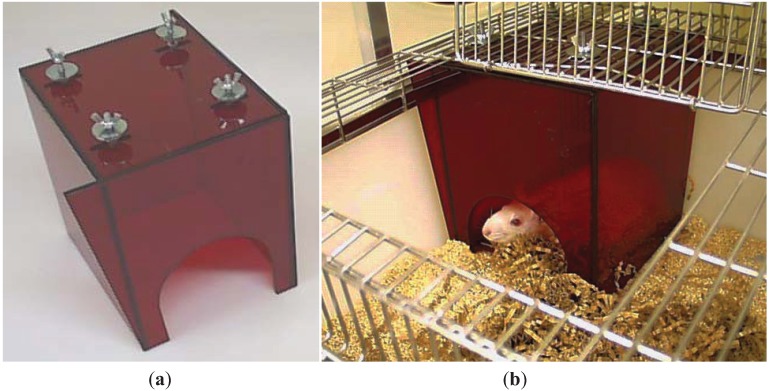
This nest box is designed for rats with external devices. (**a**) The box has a wide entrance and is bolted to the cage lid so that the animal cannot move it. The corners are extended to meet the side of the cage at the front left and back right of the box to prevent animals from squeezing between the box and cage walls. (**b**) The adapted box *in situ*.

Simply removing the enrichment item is not an appropriate solution, but in some cases there may be justification for adjusting enrichment protocols on veterinary and animal welfare grounds. For example, entanglement in nesting materials can be a real problem for rodents with head caps or cannulae, so some nesting materials would not be appropriate for them. However, it is often possible to cater for the animals’ needs in other ways, such as providing non-tangling nesting material and refuges with wide entrances that are designed so that animals cannot climb on top of them and catch exteriorised devices on the cage roof (Figure 1).

Besides concerns about potential injury, some animals may be denied enrichment (including interaction sessions with carers) in case physiological responses to the stimulation will affect data quality [31]. This is unlikely to be the case; it is more likely that isolation and boredom will cause more stress and serious welfare problems [6,20,21], with negative effects on the science.

### 2.3. Long Term Housing in the Laboratory and Reuse

Instrumented animals are often reused in biotelemetry studies, for example in order to avoid conducting surgical procedures using naïve animals. As a consequence of this, larger species such as dogs and primates may be housed in the laboratory for some time (there are legal constraints on reuse in many countries, including Member States of the European Union. These include requirements relating to the severity of the previous procedures and the health and fitness of the animal [7]). Battery life is an inevitable constraint on the duration of an animals’ stay in the laboratory, but the primary consideration should be animal welfare. Some facilities implement fixed time limits after which animals will no longer be reused, which may run to a couple of years or, exceptionally, six years or more. Others do not implement a fixed limit but regularly review the individual animals and how they respond to long term housing in the laboratory [31]. Regardless of whether time limits are in place, it is good practice to monitor animals for signs that they are becoming ‘institutionalised’ and losing the ability to cope with their environment. An appropriately tailored welfare assessment scheme, including indicators that animals are experiencing chronic adverse effects, is essential in this regard. Refining housing and care will also help to ensure that animals will be comfortable in their environment in the long term [6].

Enrichment is a husbandry refinement that can also supply additional indicators of an animal’s welfare state; for example, animals who are becoming depressed or uncomfortable may stop making properly constructed nests, or climbing onto platforms, or they may generally become less interested in their enrichment items and one another. Welfare assessment protocols should be adequately tailored to animals on long-term studies and should ensure that subtle signs of suffering can be detected before more significant welfare problems occur [25].

There is also an ethical dilemma associated with long-term housing and reuse, in which the requirement to minimise the use of naïve animals has to be balanced against setting limits on the number of times animals are re-used, and on how long they should live in research facilities. The attending veterinarian, others with animal welfare expertise and well-informed ethics or animal care and use committees can play a useful role in considering and weighing these issues, e.g., by helping to decide on humane endpoints such as behaviours that indicate chronic distress, on absolute limits on the number of times animals are reused, or on the length of time that individuals stay at the facility. Input from the attending veterinarian is an essential part of the decision-making process, and is a legal requirement in many countries [7].

### 2.4. Using Telemetered Physiological or Behavioural Data to Help Assess Wellbeing

Many variables that are monitored and recorded in biotelemetry studies are also useful in assessing animal welfare. For example, heart rate, blood pressure and body temperature can all act as indicators that animals may be experiencing pain, suffering or distress, in conjunction with other criteria such as behaviour, body weight, *etc*. Physiological data can be obtained from animals with devices in place for scientific purposes (so there are no additional harms to experimental subjects) and used to evaluate the impact of disturbances (e.g., building work), or different housing and care protocols [5]. Some examples of ways in which practitioners are using biotelemetry to assess welfare include (on the basis of an online survey conducted by the author):

- using telemetered data to help determine humane endpoints;
- reusing implanted animals in welfare studies, e.g., to provide physiological correlates with behaviour;
- using telemetered data to assess stress due to restraint and transport;
- comparing responses to different housing, enrichments and interactions with humans;
- monitoring recovery from surgery using telemetered data; and
- using physiological data to help compare the effectiveness of different analgesic agents.

Opportunities to use telemetered data to reduce the suffering and improve the welfare of the animals on the study—or other animals—should be identified and taken up wherever possible. For example, discussion between the researcher and the ethics or animal care and use committee at the project planning stage may identify the potential to use the data in this way, or this may arise at mid-term or retrospective review of a project [31].

## 3. Recommendations

Whatever the species of animal, purpose of the study or nature of the biotelemetry system, the aim should be to provide a good standard of housing and care for animals on biotelemetry studies, as for any other project. Although biotelemetry can present some challenges to refining housing and care, many of these can be overcome by flexible thinking, keeping up to date with developments and being prepared to resource new equipment and approaches, as set out in the recommendations below.

- Recognise that although biotelemetry can facilitate reduction and refinement, the technique has the potential to cause suffering—it is a ‘refinement that needs refining’. There is a need to apply a harm-benefit assessment to procedures involving biotelemetry and to ensure that these are fully refined.
- Keep up to date with technical developments such as wider availability of multi-frequency devices and smaller implants. If there are unmet technological needs that could help to refine the use of biotelemetry (e.g., multi-frequency, implantable devices for mice), communicate with device manufacturers to make them aware of the demand.
- Ensure that surgical procedures are conducted according to current good practice with respect to the competence of the surgeon, surgical approach, welfare assessment, pain management, wound closure and asepsis.
- Provide group housing for social animals used in biotelemetry studies as the default; ensure that animals are only singly housed if there is sound scientific or veterinary justification.
- If periods of single housing are unavoidable during data collection, keep these to a minimum and monitor animals carefully during reintroductions.
- Provide environmental enrichment as the default, thinking creatively about alternative approaches if some items would not be appropriate.
- Critically consider the pros and cons of automated observation systems that necessitate single housing, taking the welfare and scientific implications into account.
- Set a limit for maximum duration of laboratory housing for long term studies, with clear criteria for behavioural assessments and humane endpoints. Seek advice on this from the attending veterinarian, experienced animal technologists, and/or local ethics, animal care and use committee or animal welfare body as appropriate.
